# Bidimensional structure and measurement equivalence of the Patient Health Questionnaire-9: sex-sensitive assessment of depressive symptoms in three representative German cohort studies

**DOI:** 10.1186/s12888-021-03234-x

**Published:** 2021-05-05

**Authors:** Ana N. Tibubos, Daniëlle Otten, Daniela Zöller, Harald Binder, Philipp S. Wild, Toni Fleischer, Hamimatunnisa Johar, Seryan Atasoy, Lara Schulze, Karl-Heinz Ladwig, Georg Schomerus, Birgit Linkohr, Hans J. Grabe, Johannes Kruse, Carsten-Oliver Schmidt, Thomas Münzel, Jochem König, Elmar Brähler, Manfred E. Beutel

**Affiliations:** 1 Department of Psychosomatic Medicine and Psychotherapy, University Medical Center, Johannes Gutenberg-University Mainz, Langenbeckstraße 1, 55131 Mainz, Germany; 2 Freiburg Center of Data Analysis and Modelling, Mathematical Institute – Faculty of Mathematics and Physics, University of Freiburg, Freiburg, Germany; 3 Institute of Medical Biometry and Statistics, Faculty of Medicine and Medical Center – University of Freiburg, Freiburg, Germany; 4 Preventive Cardiology and Preventive Medicine, Department of Cardiology, University Medical Center, Johannes Gutenberg-University Mainz, Mainz, Germany; 5 Center for Thrombosis and Hemostasis, University Medical Center, Johannes Gutenberg-University Mainz, Mainz, Germany; 6 DZHK (German Center for Cardiovascular Research), Partner Site Rhine-Main, Mainz, Germany; 7 Department of Psychiatry and Psychotherapy, University Medicine Greifswald, Greifswald, Germany; 8 Department of Psychiatry and Psychotherapy, Leipzig University Medical Center, Leipzig, Germany; 9 Department of Psychosomatic Medicine and Psychotherapy, University of Gieβen and Marburg, Gieβen, Germany; 10 Helmholtz Zentrum München, German Research Center for Environmental Health, Institute of Epidemiology, Neuherberg, Germany; 11 Department of Psychosomatic Medicine and Psychotherapy, Klinikum rechts der Isar, Technische Universität München, Munich, Germany; 12 Institute for Community Management, University Medicine Greifswald, Greifswald, Germany; 13 Department of Cardiology – Cardiology I, University Medical Center, Johannes Gutenberg-University Mainz, Mainz, Germany; 14 Institute for Medical Biostatistics, Epidemiology and Informatics, University Medical Center, Johannes Gutenberg-University Mainz, Mainz, Germany

**Keywords:** Depression, Somatic dimension, Cognitive-affective dimension, Sex-differences, Regional differences

## Abstract

**Background:**

The Patient Health Questionnaire-9 (PHQ-9) has been proposed as a reliable and valid screening instrument for depressive symptoms with one latent factor. However, studies explicitly testing alternative model structures found support for a two-dimensional structure reflecting a somatic and a cognitive-affective dimension. We investigated the bidimensional structure of the PHQ-9, with a somatic (sleeping problems, fatigability, appetitive problems, and psychomotor retardation) and a cognitive-affective dimension (lack of interest, depressed mood, negative feelings about self, concentration problems, and suicidal ideation), and tested for sex- and regional-differences.

**Methods:**

We have included data from the GEnder-Sensitive Analyses of mental health trajectories and implications for prevention: A multi-cohort consortium (GESA). Privacy-preserving analyses to provide information on the overall population and cohort-specific information and analyses of variance to compare depressive, somatic and cognitive-affective symptoms between sexes and cohorts were executed in DataSHIELD. In order to determine the dimensionality and measurement invariance of the PHQ-9 we tested three models (1 factor, 2 correlated factors, and bifactor) via confirmatory analyses and performed multi-group confirmatory factor analysis.

**Results:**

Differences between sex and cohorts exist for PHQ-9 and for both of its dimensions. Women reported depressive symptoms in general as well as somatic and cognitive-affective symptoms more frequently. For all tested models an acceptable to excellent fit was found, consistently indicating a better model fit for the two-factor and bifactor model. Scalar measurement invariance was established between women and men, the three cohorts, and their interaction.

**Conclusions:**

The two facets of depression should be taken into account when using PHQ-9, while data also render support to a general factor. Somatic and cognitive-affective symptoms assessed by the PHQ-9 can be considered equivalent across women and men and between different German populations from different regions.

**Supplementary Information:**

The online version contains supplementary material available at 10.1186/s12888-021-03234-x.

## Background

The Patient Health Questionnaire-9 (PHQ-9) [1] has been proposed as a reliable and valid screening instrument for assessing depressive symptoms with one latent factor [2, 3]. It is based on DSM-IV diagnostic criteria for major depressive symptoms, the core criteria also apply to DSM-V [4]. However, studies explicitly testing alternative model structures by confirmatory factor analyses found support for a two-dimensional structure of the PHQ-9 reflecting a somatic and a cognitive-affective dimension [5–8].

This distinction is consistent with the fact that the majority of depressed patients in primary care present with somatic, rather than psychological complaints. Based on 863 participants from the Heart and Soul Study in the U.S., de Jonge and colleagues [9] identified a somatic (sleeping problems, fatigability, appetitive problems, and psychomotor agitation/ retardation) and a cognitive symptom factor (lack of interest, depressed mood, negative feelings about self, concentration problems and suicidal ideation) in the PHQ-9 using a theoretical and a factor analytical approach. Excellent model fit was observed for the proposed bidimensional PHQ-9 structure. They found that somatic, but not cognitive depressive symptoms, were associated with reduced heart rate variability, which may indicate worse cardiovascular prognosis. Subsequent papers have underscored the validity of the distinction by differential associations of the somatic (and not the cognitive) dimension. Applying the bi-factor structure in the German Gutenberg Health Study [10] only the somatic factor was associated with inflammation, vascular function and adverse life style factors (obesity, hyperlipidemia). In the Dutch Nijmegen Biomedical Study (NBS) somatic, but not cognitive items of the Beck Depression Inventory (BDI) [11] were found to be associated with atherosclerosis [12].

In general, prevalence rates of depression differ between sexes. Women’s depression rates are known to exceed the men’s rates by a factor of two across populations [13]. Yet, the question has not been settled whether this gender gap reflects sex-related differences of vulnerability, help-seeking behaviors, symptom reporting, quality of symptoms, diagnosing or gender role socialization [14]. Men may express depression behaviorally by aggression, violence, alcohol, and drug use, increasing the risk of somatic disease. Women may present with expressed anhedonia, negative emotions, sleep, appetite and weight disturbance, worthlessness and guilt.

Despite the widespread use of the PHQ-9 in more than 25 languages, only two studies have examined the underlying bidimensional symptom structure with regard to sex-differences [14, 15]. In a sample of 1168 depressed patients after spinal cord injury in the U.S., Kalpakjian and colleagues [14] analyzed item pattern loading differences between men and women. They reported low congruence when comparing the two dimensions of the PHQ-9 for both sexes, e.g. psychomotor disturbances loading on the somatic factor for women, but not for men. Using data nationally representative for the U.S. of 31,366 adult participants from the National Health and Nutrition Examination Survey (NHANES) who had filled out the PHQ-9 in one of several surveys from 2005 to 2016, Patel and colleagues [15] found a two-factor structure. However, discrepant to de Jonge and colleagues [9] and Michal and colleagues [10], the somatic dimension contained three items: sleep disturbance, fatigue and appetite changes (excluding psychomotor disturbances). The other six items were assigned to the cognitive-affective dimension. Strict measurement invariance of the bidimensional PHQ-9 held across sex. Psychometric properties would have also supported the dimension structure proposed previously [9, 10].

The factor structure of the PHQ-9 has been examined in numerous countries and languages. For most countries and subgroups, a one-factor model of the PHQ-9 was found as best fitting to the data (e.g. in a large German cohort study, in Hispanic American women [16, 17], and the general population in Hong Kong [18]. However, a systematic review of the factor structure and measurement invariance of the PHQ-9 among Portuguese speaking people found evidence for both the one- and two factor models of the PHQ-9 [19]. As underlined by previous research, not only cross-cultural [15, 16, 20], but also regional variation [21] have long been considered crucial variables of influence regarding depressive symptoms. In the eastern and western states of Germany, different political and economic systems have evolved over the 40 years from the 2nd World War to reunification in 1990. Under Eastern socialist politics of gender equality, women have been more strongly involved in education and work life, whereas traditional roles prevailed in the western states. These differences between formally eastern and western Germany potentially affect women to answer the PHQ-9 questionnaire distinctively in different regions.

Previous research has shown that the PHQ-9 is a valid screening instrument for assessing depressive symptoms with one latent factor in the general population and among women and men. Studies explicitly testing a two-dimensional structure of the PHQ-9 reflecting a somatic and a cognitive-affective dimension found a better fit to the data using two dimensions. However, only very limited sex-specific findings for this two factor-structure exist. Among other countries and subgroups, mostly a one-factor structure for PHQ-9 was found. However, a review article including studies testing factor structure and measurement invariance of the PHQ-9 for Portuguese speaking people found support for both the one- and two-factor model.

### Objective of the study

Beyond the widely common unidimensional PHQ-9 model, we investigated the bidimensional structure originally proposed by de Jonge and colleagues [9], and additionally tested a bifactor model incorporating a general factor and two specific factors [22] of the PHQ-9 for the German population taking sex differences into account. This study included data from several regions in Germany, therefore we were able to additionally test for potential socialization effects in examining the bidimensional structure of the PHQ-9 in Germany [23–25].

## Methods

### Study design and sample

The GESA consortium (GEnder-Sensitive Analyses of mental health trajectories and implications for prevention: A multi-cohort consortium) [24] included three major, ongoing, longitudinal cohorts in middle, southern and northeast Germany: the Gutenberg Health Study (GHS) [26], the Cooperative Health Research in the Augsburg Region (KORA) [27, 28] and the Study of Health in Pomerania (SHIP) [29]. These regions differ in their socioeconomic and regional characteristics [24]. Middle and southern Germany are economically stronger than northeast Germany (e.g. higher discretionary incomes and lower unemployment rates). Furthermore, these regions differ with regard to life expectancy, which is lowest in northeast Germany. Lastly, regions differ with regard to religiosity. Religiosity is higher in southern Germany and lowest in northeast Germany [30]. Based on the assessments of specific psychosocial variables, different waves of these cohorts were selected for the GESA consortium [24]. For this study GHS F1, KORA F4 and SHIP3 including data from the years 2006–2016 were selected, 304 (1.5%) (GHS 278, KORA 16 and SHIP 10) respondents with missings on all PHQ-9 items were excluded, which lead to a total sample of *N* = 19,504.

### Measures

The PHQ-9 was administered through questionnaires, either within a face-to-face interview (KORA) or filled out by the respondents (GHS, SHIP), to assess depressive symptoms over the past 2 weeks [1, 31]. Respondents indicated, on a 0–3 scale (0 = not at all; 1 = several days; 2 = more than half the days; 3 = nearly every day) the frequency with which they experienced the following symptoms: (a) anhedonia, (b) depressed mood, (c) sleep disturbance, (d) fatigue, (e) appetite changes, (f) low self-esteem, (g) concentration difficulties, (h) psychomotor disturbances, and (i) suicidal ideation. The total scores range from 0 to 27, with scores ≥10 representing clinical moderate to severe depression [1, 32]. Internal consistency of the entire questionnaire is excellent [1, 8]. Variables for a somatic depression scale and a cognitive-affective depression scale were constructed. For somatic depression the items sleep disturbance, fatigue, appetite changes, and psychomotor disturbances were combined and its total sum score ranged from 0 to 12. For cognitive-affective depression the items anhedonia, depressed mood, low self-esteem, concentration problems and suicidal ideation were combined and with a total sum score from 0 to 15.

Sociodemographic factors sex, age, years of education, marital status, living with partner, number of persons in household, employment and household income were examined. The sample consisted of 9813 females (7304 GHS; 1592 KORA; 917 SHIP) and 9691 males (7428 GHS, 1472 KORA; 791 SHIP). Age ranged from 20 to 79 (M = 55.5; SD = 11.6). For full details of the variable harmonization process, see Additional Table 1.

### Data analysis

Analyses were performed in DataSHIELD version 4.1 [33–35], which is a system for privacy-preserving analyses where individual-level data of different cohorts does not have to be pooled for joint analyses. DataSHIELD allows for analyses via several R packages, based on R-version 3.5.2 [36]. First, we performed descriptive analyses in DataSHIELD in order to provide information on the overall population and the population per cohort. Second, covariances between the items of the PHQ-9 were calculated. The covariance matrices were exported to R and used to perform confirmatory factor analysis (CFA) as well as multi-group confirmatory factor analysis (MG-CFA) with the Lavaan R-package 3.6.1 [37].

The CFAs were conducted to test the one-dimensional, two-dimensional and the bifactor model version of the PHQ-9 for women and men. In our confirmatory analyses (CFA), the variance of each latent variable was fixed to 1.0 for scaling purposes [38]. A good model fit is indicated by a non-significant (*p*-value > 0.05) or a χ2-value/df ≤ 3 [39]. In order to justify the baseline model, we considered the following fit indices: standardized root mean square residual (SRMR) root mean square error of approximation (RMSEA), Tucker-Lewis index (TLI), and comparative fit index (CFI). The results of McNeish, An, and Hancock [40] suggest adapting the levels at which good and acceptable fit are defined to the level of measurement quality, in particular the size of the factor loadings, which might lead to lower thresholds for models with better measurement. Thus, the respective cut-offs for good/acceptable/mediocre model fit are: RMSEA ≤ .060/ .080/ .100, SRMR ≤ .050/ .070/ .090, and TLI/CFI ≥ .900/ .850/ .800.

In order to determine measurement invariance of the PHQ-9 between sex, cohorts, and sex within the cohorts, we applied multi-group confirmatory factor analysis (MGCFA). In these MG-CFA’s, four models were tested sequentially, whereby each level measures an additional restriction on the model. These models are the *configural, metric, scalar* and *strict* model testing invariance for the factor structure, factor loadings, intercept values and error variance between groups. Measurement invariance testing included a series of model comparisons by applying adjusted χ^2^-difference tests [41]. A non-significant *χ2*-difference (*p* ≥ .010) indicates measurement invariance among the tested models. As the *χ2*-statistic is sensitive to sample size, we focus on the differences *ΔCFI* and *ΔRMSEA*. Values ≤ .010 indicate the invariance of the models [42, 43]. Finally, analyses of variance (ANOVA) was performed to compare women and men, the GHS, KORA and SHIP cohort and women and men within the cohorts on their scores on overall depressive symptoms, somatic and cognitive-affective depressive symptoms.

For this study, all methods were carried out in accordance with current guidelines and regulations.

## Results

### Sociodemographic characteristics

In all cohorts, depressive symptoms were more often present in women. Additionally, women scored higher than men on both the somatic and cognitive-affective dimension of depression. Male participants were on average slightly older, more often married, fulltime employed and had a higher household income. In the GHS and KORA cohort, men reported more educational years and more often lived with a partner compared to women. In SHIP these differences were not found. The number of persons in the household were only statistically significant between women and men in the GHS cohort, yet with neglectable effect size. For details, see Table 1.

**Table 1:** Sample characteristics of the GHS, KORA, and SHIP study stratified for sex (N_total_ = 19,504)

	GHS			KORA			SHIP		
	Women (*N* = 7304)	Men (*N* = 7428)	Cohen’s d	Women (*N* = 1592)	Men (*N* = 1472)	Cohen’s d	Women (*N* = 917)	Men (*N* = 791)	Cohen’s d
depression (PHQ-9 ≥ 10)			0.123***			0.124***			0.199***
*no*	6621(90.65)	6978(93.94)		1485(93.28)	1415(96.13)		850(92.69)	769(97.22)	
*yes*	683(9.35)	450(6.06)		107(6.72)	57(3.87)		67(7.31)	22(2.78)	
somatic depression	2.72 ± 2.09	2.13 ± 1.89	−0.296***	2.32 ± 2.03	1.72 ± 1.81	−0.311***	2.2 ± 1.96	1.52 ± 1.67	−0.371***
cognitive-affective depression	1.84 ± 2.00	1.51 ± 1.89	−0.170***	1.51 ± 1.82	1.13 ± 1.62	−0.220***	1.5 ± 2.00	1.05 ± 1.50	−0.252***
age (years)	54.68 ± 11.08	55.15 ± 11.09	0.042*	55.63 ± 13.11	56.6 ± 13.35	0.073*	59.23 ± 12.47	60.97 ± 13.04	0.137**
education (years)	11.27 ± 1.71	11.8 ± 1.73	0.308***	11.31 ± 2.52	12.23 ± 2.71	0.352***	12.34 ± 2.32	12.5 ± 2.55	0.066
marital status			0.280***			0.320***			0.325***
*married*	5366(73.49)	5828(78.49)		1103(69.28)	1155(78.46)		573(63.04)	577(74.07)	
*single*	690(9.45)	896(12.07)		154(9.67)	154(10.46)		103(11.33)	92(11.81)	
*divorced*	705(9.65)	550(7.41)		133(8.35)	102(6.93)		113(12.43)	73(9.37)	
*widowed*	541(7.41)	151(2.03)		202(12.69)	61(4.14)		120(13.20)	37(4.75)	
living with partner^a^			0.177***			0.237***			0.015
*no*	1623(22.23)	1140(15.35)		422(26.51)	246(16.71)		38(5.59)	35(5.11)	
*yes*	5678(77.77)	6286(84.65)		1170(73.49)	1226(83.29)		642(94.41)	650(94.89)	
number of persons in household	2.42 ± 1.11	2.52 ± 1.10	0.091***	2.48 ± 1.26	2.54 ± 1.14	0.050	2.04 ± 0.83	2.16 ± 0.83	0.145**
employment			0.854***			0.986***			0.423***
*no*	3098(42.58)	2614(35.31)		755(47.42)	620(42.12)		450(49.45)	396(50.77)	
*fulltime*	2055(28.24)	4463(60.29)		314(19.72)	796(54.08)		315(34.62)	349(44.74)	
*parttime*	1690(23.23)	214(2.89)		367(23.05)	28(1.90)		130(14.29)	22(2.82)	
*marginal*	433(5.95)	111(1.50)		156(9.8)	28(1.90)		15(1.65)	13(1.67)	
household income	3269.18 ± 2258.91	3734.43 ± 2742.23	0.185***	2251.41 ± 968.02	2465.73 ± 914.08	0.227***	2225.85 ± 1265.87	2543.41 ± 1409.02	0.238***

Note: PHQ-9 = Patient Health Questionnaire-9. Absolute (relative) numbers were used for categorical variables, mean ± standard deviation for numerical variables. To quantify the statistical significance between men and women per cohort, effect sizes (Cohen’s d) and *p*-values of the χ^2^-test or the t-test respectively, were calculated. P-values are displayed as follows: */**/*** for *p* < 0.05, *p* < 0.01 and *p* < 0.001 respectively

^a^The indicator ‘living with partner’ was extracted differently in the GHS and KORA cohort compared to the SHIP cohort, resulting in a slight overestimation of the number of people living with a partner in the SHIP cohort

### PHQ-9 factor structure

In the CFA, the one-, the correlated two-factor (cognitive-affective and a somatic dimension), and the bifactor model (general depression factor plus the two specific factors cognitive-affective and somatic depression) of the PHQ-9 were tested for the complete sample and for women and men separately. For an overview of all three tested models of the PHQ-9, see Additional Figure 1.

In order to determine the optimal factor structure of the PHQ-9, we conducted CFAs for women and men separately, for the complete sample, and stratified for cohorts (see Table 2). While the χ2/ df ≤ 3 ratio [44] indicated bad model fit in sum for all considered sample combination, other indices implied acceptable to excellent model fit for both the one- and two-factor models. Yet, the one-factor model consistently showed worse model fit compared to the correlated two-factor or bifactor model. This indicates the statistical superiority of the correlated two-factor and the bifactor model. According to the global model fit indices, on the one hand, the bifactor model turned out to fit data best. However, for two subgroups (KORA total sample and SHIP total sample) estimation problems occurred in the bifactor models. For four subgroups (men, GHS men, KORA women, SHIP women) estimation problems also occurred, but could be solved by applying the bifactor-(*S·I* – 1) model, as proposed by Eid and colleagues [45], using the fatigue item (item 4) as reference for the general factor. On the other hand, factor loadings and reliability coefficients ω are higher in the correlated two-factor model compared to the bifactor model. In the two-factor models both PHQ-9 subscales were highly correlated r = .875 overall, among women r = .883, and men r = .865 in the entire sample. In addition, sex and cohort stratified analyses emphasize the superiority of the two-factor model. The factor correlation between both dimensions varied when analyzing at cohort level: in KORA, the correlation between both dimensions was the highest overall/men/women r = .925/ .935/ .913; followed by SHIP: overall/men/women r = .898/ .866/ .902 and GHS: overall/men/women r = .860/ .850/ .870. Internal consistencies of the subscales (overall, women and men McDonald’s ω = .89–.95) and the overall scales (overall, women and men McDonald’s ω = .96) were good to excellent (see Table 3).

**Table 2:** Confirmatory factor analyses for the total sample, only women, and only men

	**Aggregated sample (GHS, KORA, SHIP)**					
	χ ^2^	df	SRMR	CFI	TLI	RMSEA
*All*						
One-factor model	2654.537	27	0.037	0.936	0.915	0.071 (0.068–0.073)
Two-factor model	2044.376	26	0.035	0.951	0.932	0.063 (0.061–0.065)
Bifactor model	940.244	18	0.022	0.978	0.955	0.051 (0.048–0.054)
*Women*						
One-factor model	1297.450	27	0.036	0.939	0.919	0.069 (0.066–0.072)
Two-factor model	1023.266	26	0.034	0.952	0.934	0.063 (0.059–0.066)
Bifactor model	450.454	18	0.022	0.979	0.959	0.049 (0.046–0.053)
*Men*						
One-factor model	1413.588	27	0.039	0.906	0.922	0.073 (0.070–0.076)
Two-factor model	1102.949	26	0.036	0.945	0.924	0.065 (0.062–0.069)
Bifactor model	693.776	19	0.029	0.966	0.935	0.061 (0.057–0.064)
	**Gutenberg Health Study (GHS)**					
	χ ^2^	df	SRMR	CFI	TLI	RMSEA
*All*						
One-factor model	1985.936	27	0.038	0.935	0.913	0.072 (0.069–0.075)
Two-factor model	1413.206	26	0.035	0.954	0.936	0.062 (0.059–0.064)
Bifactor model	649.659	18	0.021	0.980	0.960	0.049 (0.046–0.052)
*Women*						
One-factor model	962.787	27	0.038	0.937	0.915	0.071 (0.067–0.075)
Two-factor model	715.806	26	0.035	0.953	0.935	0.062 (0.058–0.066)
Bifactor model	326.319	18	0.022	0.980	0.961	0.048 (0.044–0.053)
*Men*						
One-factor model	1079.730	27	0.039	0.929	0.906	0.074 (0.070–0.078)
Two-factor model	783.712	26	0.036	0.949	0.930	0.064 (0.060–0.068)
Bifactor model	537.167	19	0.029	0.967	0.937	0.061 (0.056–0.065)
	**Cooperative Health Research in the Augsburg Region (KORA)**					
	χ ^2^	df	SRMR	CFI	TLI	RMSEA
*All*						
One-factor model	362.071	27	0.037	0.936	0.915	0.064 (0.058–0.070)
Two-factor model	337.406	26	0.036	0.941	0.918	0.063 (0.057–0.069)
Bifactor model	n/a					
*Women*						
One-factor model	202.791	27	0.038	0.936	0.915	0.064 (0.056–0.072)
Two-factor model	185.360	26	0.037	0.942	0.920	0.062 (0.054–0.071)
Bifactor model	129.692	19	0.031	0.960	0.924	0.060 (0.051–0.071)
*Men*						
One-factor model	235.487	27	0.045	0.915	0.886	0.072 (0.064–0.081)
Two-factor model	227.828	26	0.045	0.917	0.886	0.073 (0.064–0.081)
Bifactor model	76.945	18	0.024	0.976	0.952	0.047 (0.037–0.058)
	**Study of Health in Pomerania (SHIP)**					
	χ ^2^	df	SRMR	CFI	TLI	RMSEA
*All*						
One-factor model	373.300	27	0.043	0.922	0.896	0.087 (0.079–0.095)
Two-factor model	328.096	26	0.043	0.932	0.906	0.082 (0.075–0.091)
Bifactor model	n/a					
*Women*						
One-factor model	198.394	27	0.042	0.936	0.914	0.083 (0.073–0.094)
Two-factor model	171.308	26	0.041	0.945	0.924	0.078 (0.067–0.089)
Bifactor model	128.354	19	0.037	0.959	0.922	0.079 (0.067–0.092)
*Men*						
One-factor model	200.452	27	0.054	0.880	0.840	0.095 (0.084–0.107)
Two-factor model	181.152	26	0.054	0.893	0.852	0.091 (0.080–0.104)
Bifactor model	81.994	18	0.034	0.960	0.920	0.067 (0.053–0.082)

*Note*: *df* degrees of freedom, *SRMR* standardized root mean square residual, *CFI* comparative-fit-index, *TLI* Tucker-Lewis-index, *RMSEA* root mean square error of approximation, *n/a* The bifactor model did not converge

**Table 3:** Item characteristics of the Patient Health Questionnaire-9 (PHQ-9) items stratified for sex

**Total**							
			1 factor model	2 factor model	Bifactor model		
					General factor	S	CA
	Mean	SD	λ	λ	λ	λ	λ
Anhedonia (CA)	0.53	0.66	0.624	0.603	0.381		0.105
depressed mood (CA)	0.33	0.57	0.743	0.742	0.378		0.211
sleep disturbances (S)	0.92	0.92	0.532	0.532	0.447	0.160	
fatigue (S)	0.84	0.75	0.733	0.734	0.542	0.428	
appetite changes (S)	0.38	0.66	0.579	0.567	0.367	0.028	
low self-esteem (CA)	0.23	0.52	0.608	0.624	0.279		0.194
concentration difficulties (CA)	0.41	0.63	0.544	0.554	0.369		0.029
psychomotor disturbances (S)	0.16	0.46	0.439	0.434	0.223	−0.060	
suicidal ideation (CA)	0.07	0.30	0.499	0.509	0.122		0.114
Total	0.43	0.61					
Latent factor correlation				0.875			
McDonald’s ω			0.963	0.947_CA_/ 0.891 _S_	0.830	0.700	0.748
**Women**							
			1 factor model	2 factor model	Bifactor model		
					General factor	S	CA
	Mean	SD	λ	λ	λ	λ	λ
Anhedonia (CA)	0.56	0.66	0.614	0.624	0.386		0.123
depressed mood (CA)	0.39	0.60	0.720	0.743	0.408		0.293
sleep disturbances (S)	1.04	0.94	0.495	0.532	0.440	0.212	
fatigue (S)	0.92	0.77	0.685	0.733	0.524	0.425	
appetite changes (S)	0.45	0.71	0.553	0.579	0.389	0.086	
low self-esteem (CA)	0.27	0.55	0.592	0.608	0.314		0.099
concentration difficulties (CA)	0.45	0.66	0.551	0.544	0.404		−0.076
psychomotor disturbances (S)	0.17	0.47	0.443	0.439	0.234	−0.047	
suicidal ideation (CA)	0.09	0.32	0.487	0.499	0.143		0.069
Total	0.48	0.63					
Latent factor correlation				0.883			
McDonald’s ω			0.960	0.945_CA_/0.889 _S_	0.832	0.691	0.763
**Men**							
			1 factor model	2 factor model	Bifactor model		
					General factor	S	CA
	Mean	SD	λ	λ	λ	λ	λ
Anhedonia (CA)	0.50	0.66	0.578	0.583	0.370		0.099
depressed mood (CA)	0.28	0.55	0.713	0.733	0.347		0.187
sleep disturbances (S)	0.80	0.88	0.460	0.510	0.456	0.086	
fatigue (S)	0.75	0.73	0.663	0.722	0.537		
appetite changes (S)	0.32	0.59	0.514	0.539	0.320	−0.082	
low self-esteem (CA)	0.20	0.48	0.622	0.641	0.247		0.239
concentration difficulties (CA)	0.37	0.61	0.566	0.560	0.340		0.083
psychomotor disturbances (S)	0.15	0.44	0.428	0.430	0.188	−0.033	0.118
suicidal ideation (CA)	0.06	0.26	0.501	0.516	0.106		
Total	0.38	0.58					
Latent factor correlation				0.865			
McDonald’s ω			0.960	0.949_CA_/0.897 _S_	0.779	0.487	0.742

Note: SD = standard deviation, factor loadings (λ) are standardized. Index CA = cognitive-affective dimension, S = somatic dimension. Mean values are item level based and pooled for all cohorts. The sample differs for each item and deviates slightly from the general sample that was defined based on PHQ-9. The reason for this is that, in the GHS study, missing values for PHQ-9 were only allocated in case of missing values of at least three individual items, otherwise the total PHQ-9 score was calculated by implementing the mean score of the non-missing items for the missing items

Factor loadings for the three competing models were estimated for the entire sample and for subpopulations stratified by sex and cohort (see Table 3). The highest factor loadings were observed for item 2 (depressed mood – cognitive-affective) and item 4 (fatigue - somatic), irrespective of the underlying factor model solution. Thus, both items can be regarded as marker variables for each dimension.

When comparing descriptive statistics at the item level (see Table 3), the highest scores were reported for the somatic factor item 3 (sleep problems), followed by item 4 (fatigue), for women and men across cohorts. The lowest scores were reported for the cognitive-affective item 9 (suicidal ideation) followed by either item 8 (psychomotor problems) or item 6 (low self-esteem). In sum, descriptive statistic patterns regarding overall item rank orders between men and women in the three cohorts appear to be similar.

### PHQ-9 measurement invariance across sex, cohort and their interaction

In order to further evaluate the two-dimensional PHQ-9 we performed MG-CFA. Since we encountered estimation problems for subgroups when applying bifactor models, MG-CFA were tested only for the correlated two-factor model. The results are shown in Table 4. For the MGCFA including sex, the configural model had an acceptable model fit (CFI value 0.95, RMSEA value 0.06). The changes in CFI and RMSEA in the metric compared to the configural model and the scalar compared to the metric model were smaller than 0.01. This indicated that factor structures, factor loadings and intercept values are similar for women and men. In the strict model, the change in CFI was slightly above 0.01, but since the change in RMSEA was smaller than 0.01, this model was still invariant and indicated that the error variances were equal for both sexes. When analyzing the cohorts separately, results were similar. In the configural model, the CFI value was 0.95 and the RMSEA value slightly above 0.06. These values indicate an acceptable model fit. The changes in CFI and RMSEA between the metric and configural, and the scalar and the metric model did not exceed 0.01, which indicates equal factor structure, factor loadings and intercept values between the cohorts. In the strict model, the change in CFI was above 0.01, but the change in RMSEA smaller than .01, still indicating an invariant model and equal error variances between the cohorts.When testing measurement invariance for the two-dimensional PHQ-9 for sex and cohort, in the configural model, the CFI value was slightly below 0.95 and the RMSEA value slightly above 0.06. This indicated a good fit of the model. The changes in CFI and RMSEA between the metric and configural and the scalar and the metric model did respectively slightly exceed and not exceed 0.01, which indicated equal factor structure, factor loadings and intercept values between the sexes within the different cohorts. The changes in CFI and RMSEA between the strict and the scalar model were higher than 0.01. This indicated that this last restriction caused the model to fit the data worse. It implied that the error variances between the sexes in the different cohorts differed from each other. Since this difference was only present on the strict scale, one can conclude that the bidimensional structure of the PHQ-9 can be applied when measuring sex in the three different cohorts.

**Table 4:** Multi-group factor analyses for sex, cohort and sex*cohort based on the correlated two dimensions of the PHQ-9

	χ^2^	df	Δ χ 2	p	CFI	Δ CFI	RMSEA	Δ RMSEA
*Multigroup analysis - sex*								
Configural	2126.215	52	–	<.001	0.949	–	0.064	–
Metric	2170.316	59	44.101	<.001	0.948	−0.001	0.061	−0.003
Scalar	2307.861	66	137.545	<.001	0.945	−0.003	0.059	−0.002
Strict	3111.314	75	803.453	<.001	0.925	−0.020	0.064	0.005
*Multigroup analysis - cohorts*								
Configural	2149.418	78	–	<.001	0.950	–	0.064	–
Metric	2509.791	92	360.373	<.001	0.941	−0.009	0.064	0.000
Scalar	2899.773	106	389.982	<.001	0.932	−0.009	0.064	0.000
Strict	3974.251	124	1074.478	<.001	0.907	−0.025	0.069	0.005
Multigroup analysis - sex*cohort								
Configural	2357.950	156	–	<.001	0.946	–	0.066	–
Metric	2822.798	191	464.848	<.001	0.935	−0.011	0.065	−0.001
Scalar	3358.508	226	535.710	<.001	0.923	−0.012	0.065	0.000
Strict	5612.387	271	2253.879	<.001	0.868	−0.055	0.078	0.013

*Note*: *df* degrees of freedom, *CFI* comparative-Fit Index, *RMSEA* root mean square error of approximation

### Frequency and distribution of depressive symptoms

Overall, 7.1% of the respondents suffered from clinically relevant depressive symptoms. Across cohorts, women were more frequently affected (8.7%) than men (5.5%). Mean scores for somatic depression vs. cognitive-affective depression were 2.3 (SD = 1.99), respectively 1.6 (SD = 1.91) overall. Women scored higher on both PHQ-9 subscales; 2.6 (SD = 2.07) on the somatic scale (versus 2.0 (SD = 1.86) for men) and 1.8 (SD = 1.97) on the cognitive-affective scale (versus a score of 1.4 (SD = 1.82) for men) with small effect sizes. In sum, GHS participants of both sexes reported higher scores on the somatic and cognitive-affective scale compared to their KORA and SHIP counterparts, but with negligible differences. Larger differences between cohorts were observed for the somatic factor compared to the cognitive-affective factor. While differences between cohorts and sexes were significant, their interaction did not reveal significant effects. Detailed ANOVA results are displayed in Additional Table 2 (one-way ANOVA) and Additional Table 3a and b (two-way ANOVA).

## Discussion

Due to its brevity and its compatibility with the DSM-IV and DSM-V definitions of major depression, the PHQ-9 has become one of the mostly used screening measures for depressive symptoms. A bidimensional structure describing somatic, respectively cognitive-affective symptoms is clinically highly plausible establishing links to risk factors for cardiovascular disease, one of the major sequelae of depression [9, 10]. While depressive symptoms are presumed to differ between men and women, only two studies to date have compared patterns between men and women based on the PHQ-9 [14, 15].

We therefore investigated the common unidimensional model, the bidimensional structure originally proposed by de Jonge and colleagues [9], and additionally tested a bifactor model incorporating a general factor and two specific factors of the PHQ-9 for the German population taking sex differences into account. Using data of three large cohort studies from different areas in Germany, we were able to include regional variation of symptom patterns. In general, 7.1% of the respondents suffered from clinically relevant depressive symptoms. CFA revealed acceptable to excellent model fit for all three models. However, the correlated two-factor and bifactor models consistently showed better model fit than the one-factor model. Across cohorts, depressive symptoms and somatic and cognitive-affective symptoms were stronger present in women. Analyses of variance confirmed differences between sex and between cohorts for PHQ-9 and for both of its dimensions. Overall, we demonstrated factorial validity and provided psychometric data regarding the bidimensional PHQ-9 for the German population. Although our data clearly supports the incorporation of both facets of depression, the bifactor model tests also provide evidence justifying the assumption of a general depression factor. Scalar, but not strict measurement invariance were established between women and men, the three cohorts, and their interaction. Thus, we provided evidence that psychometrically meaningful interpretations of observed mean score differences when using the two PHQ-9 dimensions to compare the tested groups can be made. Somatic and cognitive-affective symptoms assessed by the PHQ-9 can be considered equivalent across women and men and between different German populations from different regions.

Thus, our data support previous sex-specific findings of Patel and colleagues [15] and differed from the findings of Kalpakjian and colleagues [14]. However, based on our two-correlated factor and bifactor model, we endorse the previous four-item scale of somatic symptoms including fatigue, appetite changes, sleep and psychomotor disturbances. The cognitive –affective dimension consists of depressed mood, low self-esteem, anhedonia, concentration difficulties and suicidal ideation. Irrespective of the underlying factor model solution, the highest factor loadings were observed for item 2 (depressed mood – cognitive-affective) and item 4 (fatigue - somatic).

A strength of the study is the large sample using of three cohorts across the life range and different living conditions, with equal proportions of men and women. Participants were recruited from the general population of the Eastern German States (SHIP), from middle (GHS) and southern Germany (KORA). Thus, cohorts differed regarding gender-related characteristics. As expected, there was a large gender gap between women and men in the western states regarding education, employment and household income. Thirty years after German reunification, there were also indicators for a gender gap in the SHIP cohort from the Eastern German states regarding household income and a higher rate of fully employed men vs. women. At the same time, more women worked full-time compared to the cohorts from the western states, there was a low rate of marginal employment among women, and women had a level of education comparable to men. Thus, findings are stable under different degrees of gender gap.

### Limitations and outlook

The empirical results reported herein should be considered in the light of some limitations. First, the interpretation is limited by a small number of external variables for validation. While we considered relevant sociodemographic differences between women and men in the analyses, we had no specific gender measure to assess sex role behavior or identity. Second, future studies should define and validate separate cut-off scores for the somatic and the cognitive-affective dimensions. Nonetheless, the use of cut-off scores to examine depression and consequently for dimensions of depression is a controversial issue. For depression, ambiguity of the optimal screening measure exists [46]. Cut-off scores can be preferable over other screening measures e.g. diagnostic algorithms [47], since accuracy is better when screening for major depression with PHQ-9. A cut-off score of ≥10 maximizes the sensitivity and specificity of the PHQ-9 in the general population [48]. Yet, compared to diagnostic criteria, the cut-off score of ≥10 for the PHQ-9 tends to overestimate prevalence of depression [49–51]. Fried and colleagues [52] argue that cut-off scores for depression should only be applied in case of confirmed uni-dimensionality and established measurement invariance. In our study, where we confirmed a multifactorial structure of the PHQ-9, a sum score should only be calculated when the constructs are highly correlated [52]. A strong latent factor correlation was present in our findings (total sample r = .875, women r = .883, men r = .865), therefore one could apply a calculated PHQ-9 sum score, which is also supported by the good internal consistency of a one-factor solution. Therefore, when screening for depression, the PHQ-9 is an adequate instrument. However, our results also emphasize that it is preferable to use a somatic and cognitive-affective dimension in epidemiological studies. Third, future studies should further test the two-dimensional structure of the PHQ-9 in other subpopulations. Our study showed consistent findings between women and men and populations from different German regions, but that could be different for other subpopulations. For example, scores of depressive symptoms based on PHQ-9 were much higher in cancer patients [53] and coronary heart disease patients [54] compared to the general population. Additionally, a similar but not identical two-dimensional structure of the PHQ-9 was identified in cancer patients [55]. Therefore, the underlying dimensional structure of the PHQ-9 could also differ in subgroups and focusing on somatic and cognitive-affective symptoms could be especially helpful in chronically physically ill patients. Our results provide a fundamental basis to examine somatic and cognitive-affective symptoms assessed by the PHQ-9 in women and men in the German population.

## Conclusions

Psychometrically meaningful interpretations of observed mean score differences when using the two PHQ-9 dimensions to compare the tested groups can be made. Somatic symptoms (fatigue, appetite changes, sleep and psychomotor disturbances) and cognitive-affective symptoms (depressed mood, low self-esteem, anhedonia, concentration difficulties and suicidal ideation) assessed by the PHQ-9 can be considered equivalent across women and men and between different German populations from different regions.

## Supplementary Information

**Additional file 1: Figure 1.** Overview of the Patient Health Questionnaire-9 (PHQ-9) models tested

**Additional file 2: Table 1.** Harmonizing demographic variables.

**Additional file 3: Table 2.** ANOVA of overall depressive symptoms, somatic, and cognitive-affective depressive symptoms stratified by cohorts or sex.

**Additional file 4: Table 3a.** Descriptive statistics of overall depressive symptoms, somatic, and cognitive-affective depressive symptoms stratified by sex and cohorts. **Table 3b.** Two-way ANOVA of depression, somatic, cognitive-affective depression stratified by cohorts, sex and cohort*sex*.*

## Data Availability

The data that support the findings of this study are available from Freiburg Center of Data Analysis and Modelling, but restrictions apply to the availability of these data, which were used under license for the current study, and so are not publicly available. Data are however available from the authors upon reasonable request and with permission of each individual cohort (GHS, KORA and SHIP). Data can only be analysed within the program DataSHIELD. This is a system for privacy-preserving analyses where individual-level data of different cohorts does not have to be pooled for joint analyses. Codes are available from DZ of the Center of Data Analysis and Modelling upon reasonable request. For this study, all methods were carried out in accordance with current guidelines and regulations.

## Abbreviations

ANOVA: Analyses of variance
CFA: Confirmatory factor analysis
CFI: Comparative fit index
GESA: GEnder-Sensitive Analyses of mental health trajectories and implications for prevention: A multi-cohort consortium
GHS: Gutenberg Health Study
KORA: Cooperative Health Research in the Augsburg Region
MCGFA: Multi-group confirmatory factor analysis
PHQ-9: Patient Health Questionnaire-9
RMSEA: Root mean square error of approximation
SHIP: Study of Health in Pomerania
SRMR: Standardized root mean square residual
TLI: Tucker-Lewis index
