# Supplementary material for: Bidimensional structure and measurement equivalence of the Patient Health Questionnaire-9: sex-sensitive assessment of depressive symptoms in three representative German cohort studies
Source: BMC Psychiatry. 2021 May 5;21:238. doi: 10.1186/s12888-021-03234-x (PMC8101182; doi:10.1186/s12888-021-03234-x)

Bidimensional structure and measurement equivalence of the Patient Health Questionnaire-9: Sex-sensitive assessment of depressive symptoms in three representative German cohort studies

Ana N. Tibubos[1], Daniëlle Otten[1], Daniela Zöller[2], Harald Binder[2,3], Philipp S. Wild[4,5,6], Toni Fleischer[7,8], Hamimatunnisa Johar[9,10], Seryan Atasoy[9,10,11], Lara Schulze[7], Karl-Heinz Ladwig[11], Georg Schomerus[8], Birgit Linkohr[10], Hans J. Grabe[7], Johannes Kruse[9], Carsten-Oliver Schmidt[12], Thomas Münzel[13,14], Jochem König[15], *Elmar Brähler[1], *Manfred E. Beutel[1].

*Shared last authorship

[1]Department of Psychosomatic Medicine and Psychotherapy, University Medical Center, Johannes Gutenberg-University Mainz, Mainz, Germany

[2]Freiburg Center of Data Analysis and Modelling, Mathematical Institute – Faculty of Mathematics and Physics, University of Freiburg, Freiburg, Germany

[3]Institute of Medical Biometry and Statistics, Faculty of Medicine and Medical Center – University of Freiburg, Freiburg, Germany

[4]Preventive Cardiology and Preventive Medicine, Department of Cardiology, University Medical Center, Johannes Gutenberg-University Mainz, Mainz, Germany

[5]Center for Thrombosis and Hemostasis, University Medical Center, Johannes Gutenberg-University Mainz, Mainz, Germany

[6]DZHK (German Center for Cardiovascular Research), Partner Site Rhine-Main, Mainz, Germany

[7]Department of Psychiatry and Psychotherapy, University Medicine Greifswald, Greifswald, Germany

[8]Department of Psychiatry and Psychotherapy, Leipzig University Medical Center, Leipzig, Germany

[9]Department of Psychosomatic Medicine and Psychotherapy, University of Gieβen and Marburg, Gieβen, Germany

[10]Institute of Epidemiology, Helmholtz Zentrum München, German Research Center for Environmental Health, Neuherberg, Germany

[11]Department of Psychosomatic Medicine and Psychotherapy, Klinikum rechts der Isar, Technische Universität München, Munich, Germany

[12]Institute for Community Management, University Medicine Greifswald, Greifswald, Germany

[13]Department of Cardiology – Cardiology I, University Medical Center, Johannes Gutenberg-University Mainz, Mainz, Germany

[14]German Center for Cardiovascular Research (DZHK), partner site Rhine-Main, Mainz, Germany

[15]Institute for Medical Biostatistics, Epidemiology and Informatics, University Medical Center, Johannes Gutenberg-University Mainz, Mainz, Germany

Correspondence:

M.Sc. Daniëlle Otten

Department of Psychosomatic Medicine and Psychotherapy

University Medical Center of the Johannes Gutenberg-University Mainz

Langenbeckstraße 1, 55131 Mainz, Germany

Phone: +49 (0)6131 17-7643

E-Mail: Danielle.Otten@unimedizin-mainz.de

Additional Figure 1. Overview of the Patient Health Questionnaire-9 (PHQ-9) models tested

1a) 1 factor model

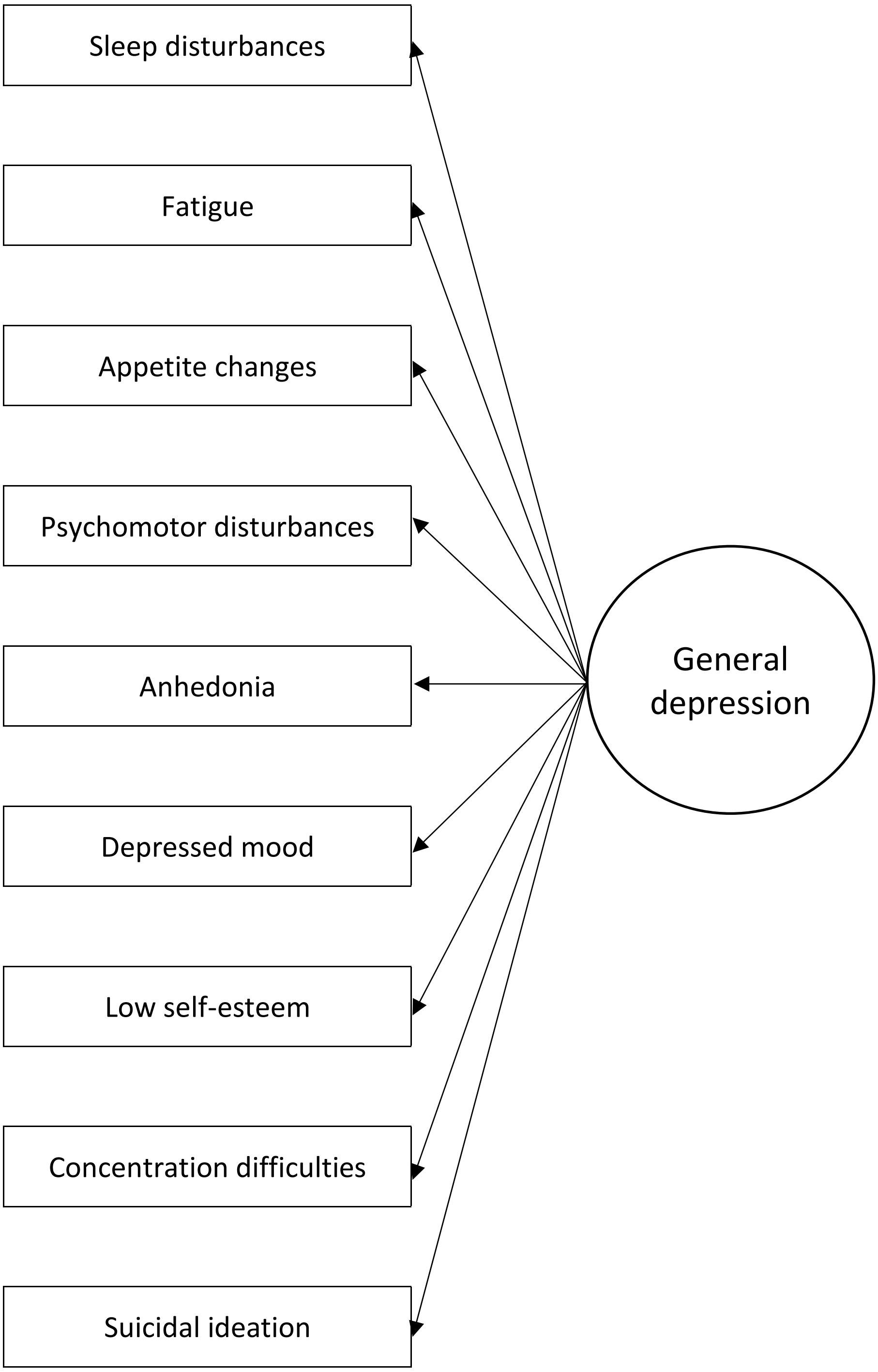

1b) Correlated 2 factor model

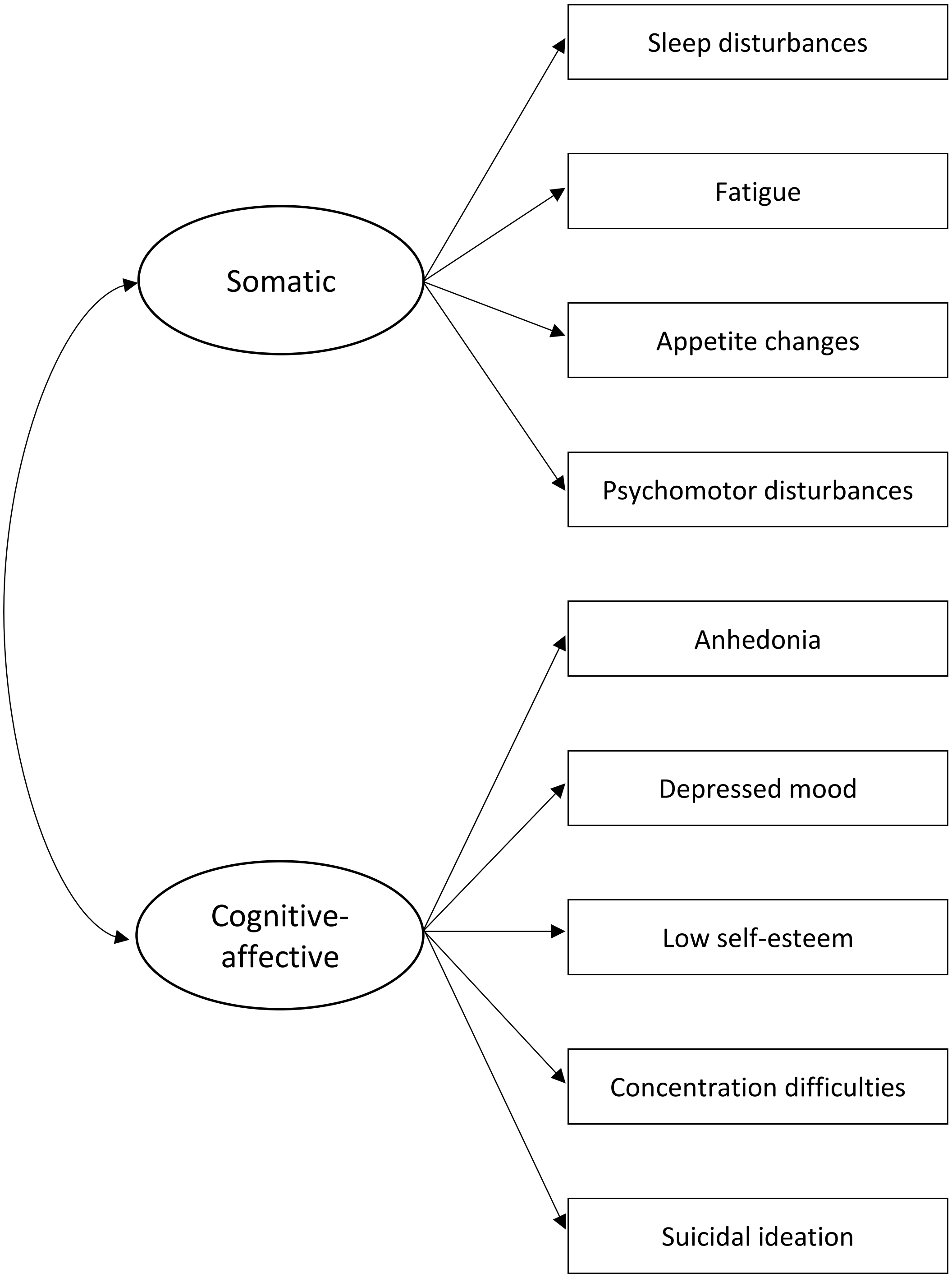

1c) Bifactor model

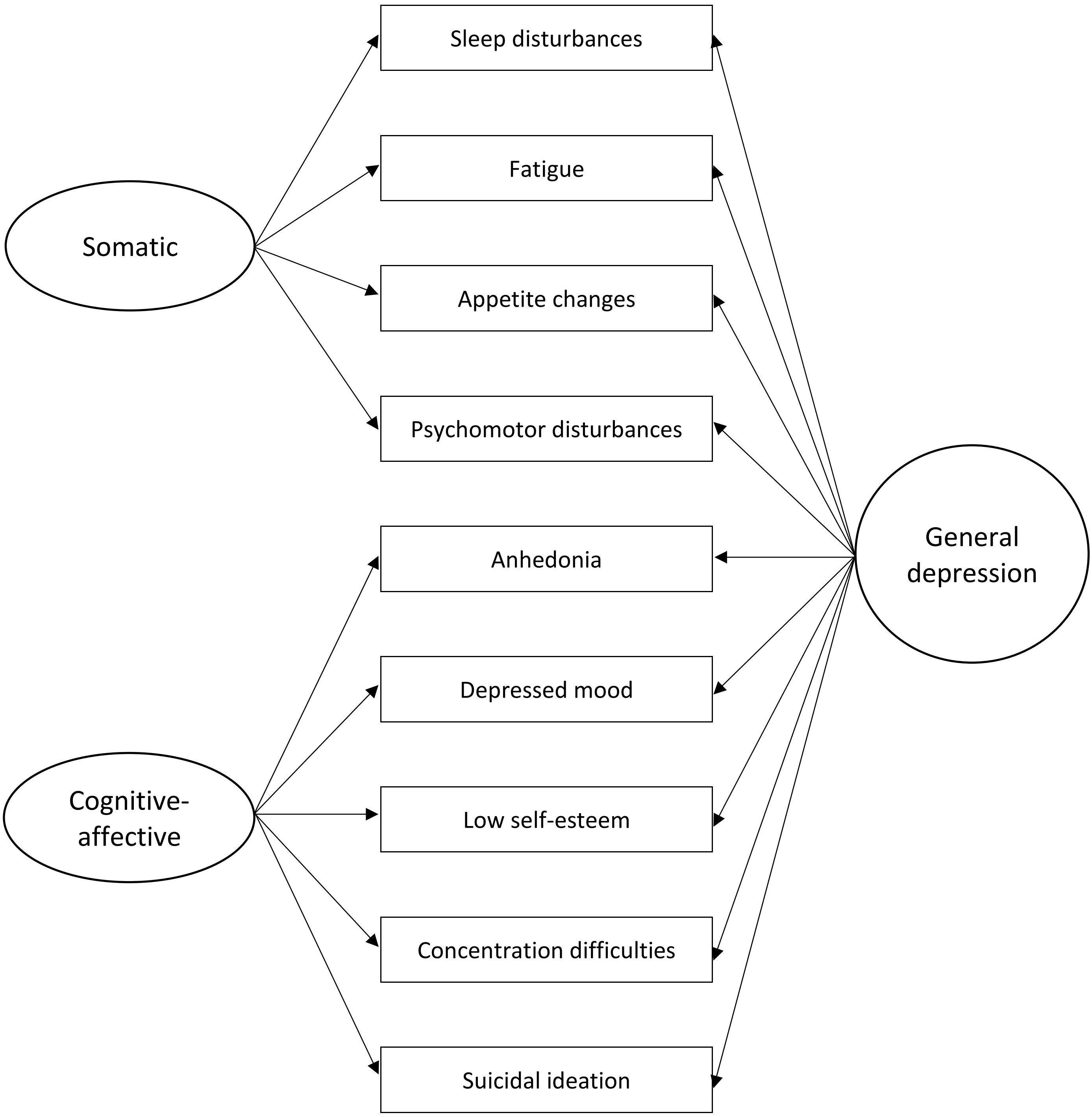

Supplement: Supplementary file 1 — Additional file 1: Figure 1. Overview of the Patient Health Questionnaire-9 (PHQ-9) models tested [file 12888_2021_3234_MOESM1_ESM.pdf]
