# Supplementary material for: Bidimensional structure and measurement equivalence of the Patient Health Questionnaire-9: sex-sensitive assessment of depressive symptoms in three representative German cohort studies
Source: BMC Psychiatry. 2021 May 5;21:238. doi: 10.1186/s12888-021-03234-x (PMC8101182; doi:10.1186/s12888-021-03234-x)
Supplement: Supplementary file 2 — Additional file 2: Table 1. Harmonizing demographic variables. [file 12888_2021_3234_MOESM2_ESM.pdf]

Bidimensional structure and measurement equivalence of the Patient Health Questionnaire-9: Sex-sensitive assessment of depressive symptoms in three representative German cohort studies

Ana N. Tibubos[1], Daniëlle Otten[1], Daniela Zöller[2], Harald Binder[2,3], Philipp S. Wild[4,5,6], Toni Fleischer[7,8], Hamimatunnisa Johar[9,10], Seryan Atasoy[9,10,11], Lara Schulze[7], Karl-Heinz Ladwig[11], Georg Schomerus[8], Birgit Linkohr[10], Hans J. Grabe[7], Johannes Kruse[9], Carsten-Oliver Schmidt[12], Thomas Münzel[13,14], Jochem König[15], *Elmar Brähler[1], *Manfred E. Beutel[1].

*Shared last authorship

[1]Department of Psychosomatic Medicine and Psychotherapy, University Medical Center, Johannes Gutenberg-University Mainz, Mainz, Germany

[2]Freiburg Center of Data Analysis and Modelling, Mathematical Institute – Faculty of Mathematics and Physics, University of Freiburg, Freiburg, Germany

[3]Institute of Medical Biometry and Statistics, Faculty of Medicine and Medical Center – University of Freiburg, Freiburg, Germany

[4]Preventive Cardiology and Preventive Medicine, Department of Cardiology, University Medical Center, Johannes Gutenberg-University Mainz, Mainz, Germany

[5]Center for Thrombosis and Hemostasis, University Medical Center, Johannes Gutenberg-University Mainz, Mainz, Germany

[6]DZHK (German Center for Cardiovascular Research), Partner Site Rhine-Main, Mainz, Germany

[7]Department of Psychiatry and Psychotherapy, University Medicine Greifswald, Greifswald, Germany

[8]Department of Psychiatry and Psychotherapy, Leipzig University Medical Center, Leipzig, Germany

[9]Department of Psychosomatic Medicine and Psychotherapy, University of Gieβen and Marburg, Gieβen, Germany

[10]Institute of Epidemiology, Helmholtz Zentrum München, German Research Center for Environmental Health, Neuherberg, Germany

[11]Department of Psychosomatic Medicine and Psychotherapy, Klinikum rechts der Isar, Technische Universität München, Munich, Germany

[12]Institute for Community Management, University Medicine Greifswald, Greifswald, Germany

[13]Department of Cardiology – Cardiology I, University Medical Center, Johannes Gutenberg-University Mainz, Mainz, Germany

[14]German Center for Cardiovascular Research (DZHK), partner site Rhine-Main, Mainz, Germany

[15]Institute for Medical Biostatistics, Epidemiology and Informatics, University Medical Center, Johannes Gutenberg-University Mainz, Mainz, Germany

Correspondence:

M.Sc. Daniëlle Otten

Department of Psychosomatic Medicine and Psychotherapy

University Medical Center of the Johannes Gutenberg-University Mainz

Langenbeckstraße 1, 55131 Mainz, Germany

Phone: +49 (0)6131 17-7643

E-Mail: Danielle.Otten@unimedizin-mainz.de

Additional Table 1: Harmonizing demographic variables

Constructed variables	Harmonisation process
Sex	Self-reported (male/ female).
Age<br>(in years)	Self-reported or derived from birthday and study date.
Education<br>(in years)	Self-reported or derived from educational degree and work educational degree. For the GHS and KORA cohort the variables highest educational level and highest professional level were combined, for the SHIP cohort a variable containing both of these aspects already existed. Years of education were assigned to the different educational, vocational and professional levels and all respondents received a score ranging from 7 to 18 years.
Marital status	Recoded into four categories married (1), not married/single (2), divorced (3), widowed (4). The KORA variable was not adapted. For the GHS cohort the categories 'registered partnership' and 'married but living separately' were added to the category married, for the SHIP cohort the category 'married, but living separately' was added to the married category.
People per household	Self-reported participant and spouse, children etc., continuous variable.
Living with partner	Included as a dichotomous variable (yes/no). Similar in the GHS and KORA cohort where respondents directly answered the question 'do you live together with your partner'. For SHIP the variable was constructed out of two questions, one relating to marital status and one related to living together with a partner. People wo answered the marital status question with 'married and living with partner' or 'married and not living with partner' were respectively assigned 'yes' and 'no' to the living together with partner variable. People wo indicated not to be married became the question 'do you live together with your partner'. Confirmation or contradiction indicated respectively 'yes' and 'no'.
Living alone	Derived from people per household (yes/ no).
Current employment	Derived from combination of employment variables, recoded into categories: not employed (0), fulltime employed (1), part-time employed (2) and marginally employed (3). The GHS and KORA variables already contained these categories, for SHIP the variable was derived from the following two questions: currently employed (yes/no) and number of hours employed. Answering 'no' to the first question was categorized as not employed; answering 'yes' to the

Constructed variables	Harmonisation process
	first question and indicating to be working 35 or more hours per week, working 15-34 hours a week and working less than 15 hours a week were categorized as respectively fulltime employment, part-time employment and marginal employment.
Household income	Included as a continuous variable. This variable was based on the three categorical household income variables in the three cohorts. For each person the mean value of the original income category was assigned. For the lowest category the lower bound was set to 0, for the highest category the upper bound was calculated by summing the lowest value and the difference (mean value of the previous category minus the lowest value in the previous category). This new metric variable with the mean value of income category per person was included in the project.
