# Supplementary material for: Bidimensional structure and measurement equivalence of the Patient Health Questionnaire-9: sex-sensitive assessment of depressive symptoms in three representative German cohort studies
Source: BMC Psychiatry. 2021 May 5;21:238. doi: 10.1186/s12888-021-03234-x (PMC8101182; doi:10.1186/s12888-021-03234-x)
Supplement: Supplementary file 3 — Additional file 3: Table 2. ANOVA of overall depressive symptoms, somatic, and cognitive-affective depressive symptoms stratified by cohorts or sex. [file 12888_2021_3234_MOESM3_ESM.pdf]

Bidimensional structure and measurement equivalence of the Patient Health Questionnaire-9: Sex-sensitive assessment of depressive symptoms in three representative German cohort studies

Ana N. Tibubos[1], Daniëlle Otten[1], Daniela Zöller[2], Harald Binder[2,3], Philipp S. Wild[4,5,6], Toni Fleischer[7,8], Hamimatunnisa Johar[9,10], Seryan Atasoy[9,10,11], Lara Schulze[7], Karl-Heinz Ladwig[11], Georg Schomerus[8], Birgit Linkohr[10], Hans J. Grabe[7], Johannes Kruse[9], Carsten-Oliver Schmidt[12], Thomas Münzel[13,14], Jochem König[15], *Elmar Brähler[1], *Manfred E. Beutel[1].

*Shared last authorship

[1]Department of Psychosomatic Medicine and Psychotherapy, University Medical Center, Johannes Gutenberg-University Mainz, Mainz, Germany

[2]Freiburg Center of Data Analysis and Modelling, Mathematical Institute – Faculty of Mathematics and Physics, University of Freiburg, Freiburg, Germany

[3]Institute of Medical Biometry and Statistics, Faculty of Medicine and Medical Center – University of Freiburg, Freiburg, Germany

[4]Preventive Cardiology and Preventive Medicine, Department of Cardiology, University Medical Center, Johannes Gutenberg-University Mainz, Mainz, Germany

[5]Center for Thrombosis and Hemostasis, University Medical Center, Johannes Gutenberg-University Mainz, Mainz, Germany

[6]DZHK (German Center for Cardiovascular Research), Partner Site Rhine-Main, Mainz, Germany

[7]Department of Psychiatry and Psychotherapy, University Medicine Greifswald, Greifswald, Germany

[8]Department of Psychiatry and Psychotherapy, Leipzig University Medical Center, Leipzig, Germany

[9]Department of Psychosomatic Medicine and Psychotherapy, University of Gieβen and Marburg, Gieβen, Germany

[10]Institute of Epidemiology, Helmholtz Zentrum München, German Research Center for Environmental Health, Neuherberg, Germany

[11]Department of Psychosomatic Medicine and Psychotherapy, Klinikum rechts der Isar, Technische Universität München, Munich, Germany

[12]Institute for Community Management, University Medicine Greifswald, Greifswald, Germany

[13]Department of Cardiology – Cardiology I, University Medical Center, Johannes Gutenberg-University Mainz, Mainz, Germany

[14]German Center for Cardiovascular Research (DZHK), partner site Rhine-Main, Mainz, Germany

[15]Institute for Medical Biostatistics, Epidemiology and Informatics, University Medical Center, Johannes Gutenberg-University Mainz, Mainz, Germany

Correspondence:

M.Sc. Daniëlle Otten

Department of Psychosomatic Medicine and Psychotherapy

University Medical Center of the Johannes Gutenberg-University Mainz

Langenbeckstraße 1, 55131 Mainz, Germany

Phone: +49 (0)6131 17-7643

E-Mail: Danielle.Otten@unimedizin-mainz.de

Additional Table 2. ANOVA of overall depressive symptoms, somatic, and cognitive-affective depressive symptoms stratified by cohorts or sex

**Overall depressive symptoms**						
Group comparisons		Mean	SD	N	p	Effect size
Cohort	GHS	4.09	3.56	14732	<.0001	0.009
	KORA	3.36	3.30	3064		
	SHIP	3.18	3.33	1708		
Sex	women	4.35	3.63	9813	<.0001	0.017
	men	3.43	3.29	9691		
Pairwise comparisons		Mean diff	SD	LB	UB	
Cohort	GHS-KORA	0.21	0.02	0.17	0.25	
	GHS-SHIP	0.26	0.03	0.21	0.31	
	KORA-SHIP	0.05	0.03	-0.01	0.11	
Sex	women-men	0.26	0.01	0.24	0.29	
**Somatic subscale**						
Group comparisons		Mean	SD	N	p	Effect size
Cohort	GHS	2.42	2.01	14360	<.0001	0.009
	KORA	2.03	1.95	3066		
	SHIP	1.89	1.87	1711		
Sex	women	2.61	2.07	9594	<.0001	0.022
	men	2.02	1.86	9543		
Pairwise comparisons		Mean diff	SD	LB	UB	
Cohort	GHS-KORA	0.20	0.02	0.16	0.23	
	GHS-SHIP	0.27	0.03	0.22	0.32	
	KORA-SHIP	0.07	0.03	0.01	0.13	
Sex	women-men	0.30	0.01	0.27	0.33	
**Cognitive-affective subscale**						
Group comparisons		Mean	SD	N	p	Effect size
Cohort	GHS	1.68	1.95	14300	<.0001	0.007
	KORA	1.33	1.74	3064		
	SHIP	1.30	1.80	1711		
Sex	women	1.76	1.97	9570	<.0001	0.008
	men	1.41	1.82	9505		
Pairwise comparisons		Mean diff	SD	LB	UB	
Cohort	GHS-KORA	0.18	0.02	0.14	0.22	
	GHS-SHIP	0.20	0.03	0.15	0.25	
	KORA-SHIP	0.02	0.03	-0.04	0.08	
Sex	women-men	0.18	0.01	0.15	0.21	

Note: Mean diff = standardized mean differences, SD = standard deviation, LB = Lower bound & UB = Upper bound of 95% confidence interval, effect size = partial eta squared (η_p^2), GHS = Gutenberg Health Study, KORA = Cooperative Health Research in the Augsburg Region, SHIP = Study of Health in Pomerania.
