# Supplementary material for: Bidimensional structure and measurement equivalence of the Patient Health Questionnaire-9: sex-sensitive assessment of depressive symptoms in three representative German cohort studies
Source: BMC Psychiatry. 2021 May 5;21:238. doi: 10.1186/s12888-021-03234-x (PMC8101182; doi:10.1186/s12888-021-03234-x)
Supplement: Supplementary file 4 — Additional file 4: Table 3a. Descriptive statistics of overall depressive symptoms, somatic, and cognitive-affective depressive symptoms stratified by sex and cohorts. Table 3b. Two-way ANOVA of depression, somatic, cognitive-affective depression stratified by cohorts, sex and cohort*sex. [file 12888_2021_3234_MOESM4_ESM.pdf]

Bidimensional structure and measurement equivalence of the Patient Health Questionnaire-9: Sex-sensitive assessment of depressive symptoms in three representative German cohort studies

Ana N. Tibubos[1], Daniëlle Otten[1], Daniela Zöller[2], Harald Binder[2,3], Philipp S. Wild[4,5,6], Toni Fleischer[7,8], Hamimatunnisa Johar[9,10], Seryan Atasoy[9,10,11], Lara Schulze[7], Karl-Heinz Ladwig[11], Georg Schomerus[8], Birgit Linkohr[10], Hans J. Grabe[7], Johannes Kruse[9], Carsten-Oliver Schmidt[12], Thomas Münzel[13,14], Jochem König[15], *Elmar Brähler[1], *Manfred E. Beutel[1].

*Shared last authorship

[1]Department of Psychosomatic Medicine and Psychotherapy, University Medical Center, Johannes Gutenberg-University Mainz, Mainz, Germany

[2]Freiburg Center of Data Analysis and Modelling, Mathematical Institute – Faculty of Mathematics and Physics, University of Freiburg, Freiburg, Germany

[3]Institute of Medical Biometry and Statistics, Faculty of Medicine and Medical Center – University of Freiburg, Freiburg, Germany

[4]Preventive Cardiology and Preventive Medicine, Department of Cardiology, University Medical Center, Johannes Gutenberg-University Mainz, Mainz, Germany

[5]Center for Thrombosis and Hemostasis, University Medical Center, Johannes Gutenberg-University Mainz, Mainz, Germany

[6]DZHK (German Center for Cardiovascular Research), Partner Site Rhine-Main, Mainz, Germany

[7]Department of Psychiatry and Psychotherapy, University Medicine Greifswald, Greifswald, Germany

[8]Department of Psychiatry and Psychotherapy, Leipzig University Medical Center, Leipzig, Germany

[9]Department of Psychosomatic Medicine and Psychotherapy, University of Gieβen and Marburg, Gieβen, Germany

[10]Institute of Epidemiology, Helmholtz Zentrum München, German Research Center for Environmental Health, Neuherberg, Germany

[11]Department of Psychosomatic Medicine and Psychotherapy, Klinikum rechts der Isar, Technische Universität München, Munich, Germany

[12]Institute for Community Management, University Medicine Greifswald, Greifswald, Germany

[13]Department of Cardiology – Cardiology I, University Medical Center, Johannes Gutenberg-University Mainz, Mainz, Germany

[14]German Center for Cardiovascular Research (DZHK), partner site Rhine-Main, Mainz, Germany

[15]Institute for Medical Biostatistics, Epidemiology and Informatics, University Medical Center, Johannes Gutenberg-University Mainz, Mainz, Germany

Correspondence:

M.Sc. Daniëlle Otten

Department of Psychosomatic Medicine and Psychotherapy

University Medical Center of the Johannes Gutenberg-University Mainz

Langenbeckstraße 1, 55131 Mainz, Germany

Phone: +49 (0)6131 17-7643

E-Mail: Danielle.Otten@unimedizin-mainz.de

Additional Table 3a. Descriptive statistics of overall depressive symptoms, somatic, and cognitive-affective depressive symptoms stratified by sex and cohorts

Overall depressive symptoms				
Sex	Cohort	Mean	SD	N
women	GHS	4.54	3.67	7304
	KORA	3.84	3.45	1592
	SHIP	3.70	3.63	917
men	GHS	3.64	3.38	7428
	KORA	2.85	3.04	1472
	SHIP	2.57	2.83	791
**Somatic subscale**				
Sex	Cohort	Mean	SD	N
women	GHS	2.72	2.09	7084
	KORA	2.32	2.03	1592
	SHIP	2.20	1.97	918
men	GHS	2.13	1.89	7276
	KORA	1.72	1.82	1474
	SHIP	1.52	1.67	793
**Cognitive-affective subscale**				
Sex	Cohort	Mean	SD	N
women	GHS	1.84	2.00	7059
	KORA	1.51	1.82	1592
	SHIP	1.51	2.00	919
men	GHS	1.51	1.89	7241
	KORA	1.13	1.62	1472
	SHIP	1.05	1.50	792

Note: SD = standard deviation, GHS = Gutenberg Health Study, KORA = Cooperative Health Research in the Augsburg Region, SHIP = Study of Health in Pomerania.

Additional Table 3b. Two-way ANOVA of depression, somatic , cognitive-affective depression stratified by cohorts, sex and cohort*sex

**Overall depressive symptoms**					
	df	MS	F	p	Effect size
cohorts	2	766.38	63.699	<.0001	0.006
sex	1	2317.12	192.590	<.0001	0.010
cohorts*sex	2	10.04	0.835	0.434	0.000
within groups	19498	12.03			
total	19503				
**Somatic subscale**					
	df	MS	F	p	Effect size
cohorts	2	256.50	66.127	<.0001	0.004
sex	1	891.86	229.929	<.0001	0.005
cohorts*sex	2	1.88	0.485	0.616	0.000
within groups	19131	3.88			
total	19136				
**Cognitive-affective subscale**					
	df	MS	F	p	Effect size
cohorts	2	145.30	35.686	<.0001	0.004
sex	1	347.27	40.293	<.0001	0.005
cohorts*sex	2	2.77	96.305	0.463	0.000
within groups	19069	3.61	0.769		
total	19074				

Note: Df = degree of freedom, MS = Mean squares, effect size = partial eta squared (η_p^2)
